# Differential Protein Content between Fresh and Freeze-Dried Plasma Rich in Growth Factors Eye Drops

**DOI:** 10.3390/biom12091215

**Published:** 2022-09-01

**Authors:** Eduardo Anitua, Ander Pino, Mikel Azkargorta, Felix Elortza, Jesús Merayo-Lloves, Francisco Muruzabal

**Affiliations:** 1 BTI—Biotechnology Institute, 01007 Vitoria, Spain; 2 Research and Development Department, University Institute for Regenerative Medicine and Oral Implantology—UIRMI (UPV/EHU-Fundación Eduardo Anitua), 01007 Vitoria, Spain; 3 Proteomics Platform, CIC bioGUNE, CIBERehd, ProteoRed-ISCIII, Bizkaia Science and Technology Park, 48160 Derio, Spain; 4 Fundación de Investigación Oftalmológica, Instituto Oftalmológico Fernández-Vega, 33012 Oviedo, Spain

**Keywords:** plasma rich in growth factors, PRGF, freeze-dried, lyophilization, proteomic, ocular surface, eye-drops, ocular disorders

## Abstract

The purpose of this study was to analyze the proteomic composition of plasma rich in growth factors eye drops (PRGF) in comparison to lyophilized PRGF eye drops (PRGF lyo). The differential protein expression of keratocyte (HK) cells after PRGF or PRGF lyo treatment was also determined. Blood from different donors was collected and processed to obtain PRGF and PRGF lyo eye drops. Then, HK cells were treated with both formulations. A proteomic analysis was performed to evaluate the differential proteomic profile between PRGF and PRGF lyo, and the differential protein expression by HK cells after treatment with both blood-derived products. About 280 proteins were detected between both blood-derived formulation, with only 8 of them reaching significant differences. Furthermore, 101 out of 3213 proteins showed statistically significant deregulation in HK cells after treatment with PRGF or PRGF lyo. Gene Ontology analysis showed that these significant deregulated proteins were involved in 30 functional processes. However, the Ingenuity Pathway Analysis showed that no significant differences were found in any of the identified processes. In summary, the present study show that no significant differences were found in the proteomic profile or in the signaling pathways activation in HK cells between PRGF and PRGF lyo.

## 1. Introduction

The incidence of the different ocular surface disorders has been increasing along the last years [1]. The functional regeneration of the damaged ocular tissue is necessary and essential to recover the complete eye function and improve the patient life quality [2]. Most of the ocular surface disorders are commonly treated with artificial tears, but these treatments lack the characteristics of the natural tears [3]. Furthermore, they often contain additives that can potentially induce toxic or allergic reactions [4]. Several other therapies including anti-inflammatory drugs are frequently used for the treatment of ocular surface disorders showing symptom improvements, but their continued use could induce some drawbacks like ocular burning or increased ocular pressure [5,6,7].

Over the last three decades, blood derivative products like autologous serum (AS) and platelet-rich plasmas (PRP) have been developed for the treatment of different ocular pathologies [8,9,10]. The features of these products are similar to the natural tears such as their regenerative, lubrication, antimicrobial and anti-inflammatory properties. In addition, the characteristics of the blood-derivative products is very similar to the natural tears in terms of osmolarity, pH and many proteins which are involved in tissue regeneration [6,11,12]. These blood-derivative products have been successfully used for the treatment of several ocular surface disease like dry eye disease, persistent epithelial defects, or corneal ulcers [8]. Nonetheless, the differential composition between both types of blood-derived products due to their different manufacturing protocol has been widely demonstrated [13,14]. As a consequence, different preclinical and clinical results have been obtained when both products have been used [15,16,17,18,19].

Plasma rich in growth factors (PRGF) eye drops is a type of PRP with specific characteristics including moderate platelet concentration, platelet activation, and lack of leukocytes [15,20]. PRGF eye drops have been used for the treatment of several ocular surface diseases such as corneal epithelial defects, dry eye, neurotrophic keratitis and graft versus host diseases among others, obtaining encouraging clinical outcomes [21].

Ocular surface disorders require medium- or long-term treatment because of their usual chronic condition. Therefore, it is necessary that treatments maintain their stability for long periods of time in order to be used daily for months. Several studies have demonstrated the safety and stability of PRGF eye drops stored for up to twelve months under low temperatures [22]. However, it implies that their long-term storage is dependent on a cold chain (−20 °C storage to keep it for a long period of time and +4 °C or room temperature during its use) [22,23,24].

On the other hand, although PRGF eye drops are commonly used in an autologous manner for the treatment of ocular pathologies, allogeneic products could be an interesting alternative to be used in those patients who are not suitable to be donors due to systemic inflammatory diseases, age, and other types of disorders or comorbidities [25]. 

Lyophilization of both types of products, autologous and allogeneic eye drops, could be an alternative to achieve longer shelf-life for both products avoiding a cold chain dependence. Recent preclinical studies have shown that PRGF eye drops maintain their biological properties after undergoing a freeze-drying process without the use of lyoprotectans like trehalose [26]. Furthermore, freeze-dried PRGF eye drops preserve the main growth factors and their biological activity for up to 3 months after storage at room temperature or 4 °C [27]. 

Although the stability of the main growth factors involved in ocular tissue regeneration has been studied after PRGF lyophilization, PRGF eye drops contain a much wider range of proteins and growth factors [13], then a deeper study is required to analyze the protein content of PRGF eye drops after a freeze-drying process. In addition, the freeze-drying process could modify the protein structures due to the low temperature and the higher concentration to which the sample is subjected during the freezing time [28]. And then, some lyoprotectants like trehalose are usually added to the product to avoid protein modifications [29]. However, the PRGF lyo used for the present study was obtained without the addition of any lyoprotectant. Therefore, this highlights the desirability of carrying out further PRGF lyo proteomic analysis to assess possible proteomic modifications occurred during the freeze-drying process. Then, the purpose of the present work was to characterize the proteomic composition of plasma-rich in growth factors after a freeze-drying process (PRGF lyo) regarding fresh PRGF (PRGF). In addition, the protein deregulation of HK cells after treatment with PRGF or PRGF lyo has been also determined.

## 2. Materials and Methods

### 2.1. Plasma Rich in Growth Factors Preparations

**Figure 1:**
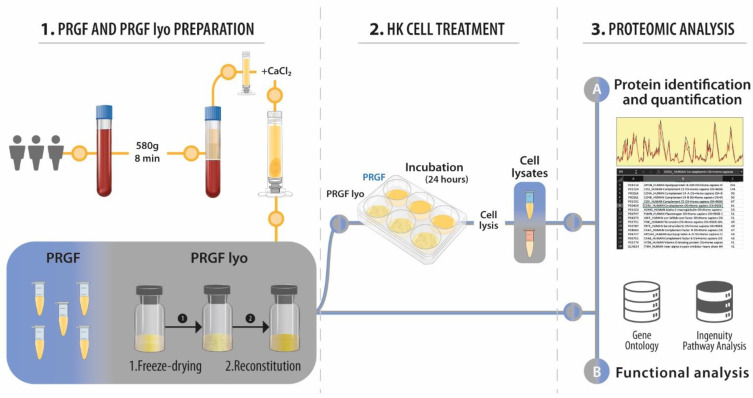
Schematic workflow of the proteomic study of the PRGF and PRGF lyo formulations. The study was divided into three parts; in the first part, the two blood derivatives, PRGF and PRGF lyo, were obtained. In the second part, HK cells were incubated with both blood derived products to analyze by proteomic techniques their response to each formulation. Finally, both PRGF and PRGF lyo were analyzed by the same proteomic techniques.

Blood from three healthy male donors was drawn off after informed consent into 9-mL tubes with 3.8% (*wt*/*v*) sodium citrate collection tubes. The study was performed following the principles of the Declaration of Helsinki. The process to obtain plasma rich in growth factors (PRGF) and the freeze-dried PRGF eye drops (PRGF lyo) was analogous to that described previously by our group [26]. PRGF from different donors was individually analyzed (not pooled). Figure 1 shows the whole workflow of the present study.

### 2.2. Cells

Human corneal keratocytes (HK) (ScienCell Research Laboratories, San Diego, CA, USA) was used to carry out the different assays of the present study. HK cells were cultured as described in previously published studies [30]. 

Keratocyte cells were seeded in a 6-well plate with serum-free medium supplemented with 20% (*v*/*v*) of the different treatments (PRGF and PRGF lyo) obtained from the three donors. Then, HK cells were incubated for 24 h. After that, culture media were removed, and cells were rinsed with PBS. Then, 400 μL of cell lysis buffer consisting of 7 M urea, 2 M thiourea, 4% CHAPS was added to each well to obtain the cellular proteins. Samples were incubated for 30 min at room temperature under agitation and digested following the filter-aided FASP protocol described by Wisniewski et al. [31] with minor modifications. Trypsin was added to a trypsin:protein ratio of 1:10, and the mixture was incubated overnight at 37 °C, dried out in a RVC2 25 speedvac concentrator (Christ, Osterode am Harz, Germany), and resuspended in 0.1% FA.

### 2.3. Proteomic Analysis

The dataset of PRGF samples used for the comparison with PRGF lyo were previously employed to obtain the results of a study published previously, in which the PRGF dataset were compared with the dataset obtained using an autologous serum formulation [13]. 

The process by which proteomic analysis was carried out was essentially as described in detail previously [13].

MaxQuant software (Max-Planck Institute for Biochemistry, Martinsried, Germany, 1.6.5.0 version) using default settings was utilized for protein identification and quantification [32]. Searches were carried out against a database consisting of human protein entries (Uniprot/Swissprot), with precursor and fragment tolerances of 20 ppm and 0.05 Da. Only proteins identified with at least two peptides at FDR < 1% were considered for further analysis. Data (LFQ intensities) was loaded onto Perseus platform (Max-Planck Institute for Biochemistry, Martinsried, Germany, 1.5.1.5 version) [33] and further processed (log2 transformation, imputation) before statistical analysis (Student’s *t*-test).

### 2.4. Functional Analysis

DAVID online tool (http://david.abcc.ncifcrf.gov/summary.jsp (accessed on 7 October 2020)) was used to analyze the enrichment of Gene Ontology (GO) terms [34,35]. DAVID is a GO Term annotation and enrichment analysis tool used to highlight the most relevant GO terms associated with a given gene list. Fisher exact test was used to analyze whether the proportion of genes related to certain GO terms or categories differed significantly between the dataset and the background. Only were considered for comparison and discussion those GO Terms enriched with a *p* value < 0.05.

Ingenuity Pathway Analysis (IPA, QIAGEN Redwood City, www.qiagen.com/ingenuity (accessed on 7 October 2020) was used to analyze the functions in which the different identified proteins were related. A Fisher’s exact test (*p*-value < 0.05 was considered significant) was used to calculate the probability by which proteins in the data set associated with a particular canonical pathway, functional network or upstream regulator was not due to chance alone. Activation z-score reflects whether a biological function is in an activated (positive values) or inactivated (negative values) state, based on the knowledge of the relation between the effectors and their target molecules.

## 3. Results

### 3.1. Proteomic Characterization of Blood-Derivative Products

**Figure 2:**
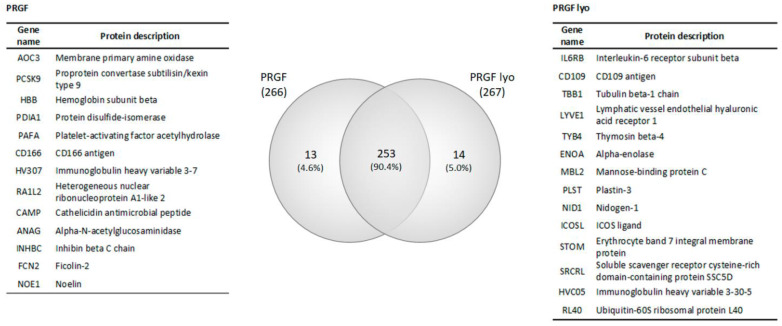
Venn diagram comparison of two blood–derived formulations and the list of proteins specific for PRGF and PRGF lyo.

PRGF preparations showed a mean platelet enrichment of 2.2-fold over the platelet concentration in peripheral blood. In addition, no detectable levels of leucocytes were observed in any of the PRGF preparations. PRGF and PRGF lyo samples obtained from the three different donors were analyzed for differential protein expression. A total of 280 different proteins were detected between both types of formulations. Supplementary File S1 contains the complete list of proteins detected in these formulations, and their relative expression. Figure 2 shows the Venn diagram where the intersection of the total proteins from the two blood-derived products (PRGF and PRGF lyo) can be observed. In summary, the number of proteins identified was 266 in PRGF eye drops and 267 in PRGF lyo eye drops. Of the total proteins identified in each formulation, 253 proteins (90.4%) were shared by both blood-derived products, while 13 (4.6%) were only related to the PRGF formulation and 14 (5.0%) to the PRGF lyo. Supplementary File S1 contains the lists of shared or formulation-specific proteins.

**Figure 3:**
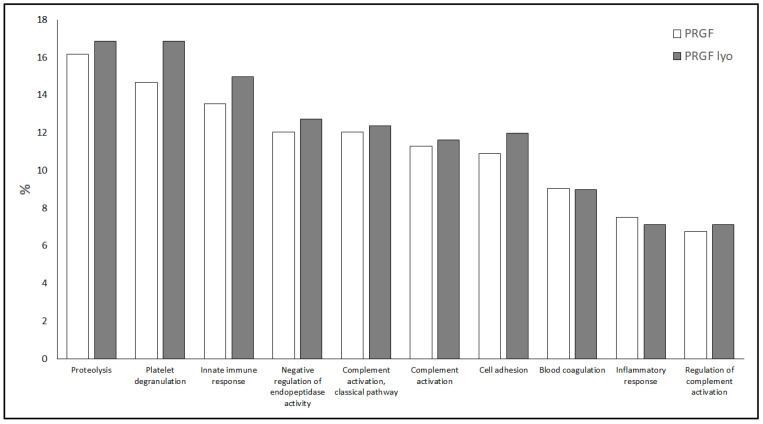
The ten most abundant Gene Ontology terms identified in PRGF and PRGF lyo formulations.

A Gene Ontology (GO) analysis was performed to evaluate the functional processes in which these proteins participate. The results of the GO analysis showed that between both blood-derived products (PRGF and PRGF lyo) a total of 236 different GO terms were revealed. Supplementary File S1 contains the lists of all GO terms obtained from each formulation. The ten most abundant GO terms found between both formulations are represented in the Figure 3. They may be grouped in 3 main processes: (i) 3 of them like proteolysis, negative regulation of endopeptidase activity and cell adhesion GO terms may be related to the cellular activity; (ii) 5 GO terms out of 10 such as innate immune response, complement activation (classical pathway), complement activation, inflammatory response and regulation of complement activation are likely related with the immune response; and finally, (iii) the last 2 GO terms: platelet degranulation and blood coagulation are related to the platelet function. As it is shown in Figure 2, the percentage of deregulated proteins involved in all this GOs are similar for both blood-derivative products. 

The relative quantitative proteomics analysis carried out to compare the protein composition of PRGF and PRGF lyo showed that a total of 257 proteins were identified with at least two different peptides, of which 8 showed significant differences (*p* < 0.05 and a ratio > 1.5 in either direction) between both formulations (Supplementary File S2). These proteins were selected for further analysis (Table 1).

The GO analysis carried out with these significantly deregulated proteins showed that they could be involved in different biological processes (Table 2). However, IPA analysis showed no significant differences in any of the processes identified (Supplementary File S3).

### 3.2. Proteomic Characterization of HK Cells Treated with Blood-Derivative Products

Protein samples isolated from HK cells treated with both types of eye drops (PRGF and PRGF lyo) obtained from the three different donors were analyzed for differential expression. The results obtained showed that out of a total of 3215 proteins identified, 101 showed statistically significant differences (Supplementary File S4). These significant deregulated proteins were used to a Gene Ontology (GO) analysis to characterize the functional processes where these proteins are involved in. GO term analysis showed that 30 out of 41 GO terms identified were significantly enriched (Supplementary File S4). 

Ingenuity pathways analysis (IPA) was carried out to evaluate the functional processes in which the deregulated proteins are involved. IPA showed that no significant differences were observed between the proteins deregulated in the HK cells after treatment with PRGF or PRGF lyo (Supplementary File S5).

## 4. Discussion

Several blood-derivative products have been developed along the last three decades to enhance tissue regeneration in different ocular surface diseases. Diverse protocols and procedures have been developed to obtain these type of blood derivatives. One of the common steps to obtain any of these blood-derived products is the withdrawal of a small volume of the patient own-blood, thus obtaining an autologous therapy [21]. However, in some cases, it is not possible to obtain an autologous product because some patients are not suitable to be donors due to certain health or physical conditions like systemic inflammatory diseases, age, and other types of disorders or comorbidities [25]. Then, allogeneic products could be an interesting alternative to be used in these patients. In the present study, PRGF and PRGF lyo were treated as independent samples to analyze not only differences between treatments, but also interindividual alterations. However, when working with allogenic therapies, pooled PRP should be considered. As previously reported by other authors, PRP preparation techniques for allogenic applications such as apheresis may also be used for the standardization and use of platelet derived products in tissue regeneration [36]. PRP obtained by these techniques can be collected in large amounts in donor centers and has the advantage of being technically standardized and reducing the inherent biological variability among donors.

On the other hand, different ocular surface disorders are usually chronic pathologies, and it makes necessary that the therapies used for their treatment must be stored for long periods of time. Until recently, blood-derived products like PRGF eye drops had to be stored at low temperatures (−20 °C) for long periods of time to be used for the treatment of ocular surface diseases. So, this made the patient dependent on the cold chain for the storage of this type of treatment. However, recent studies have demonstrated that PRGF eye drops can be freeze-dried maintaining the main growth factors that are involved in ocular surface tissue regeneration [26]. In the present study, the proteomic profile of fresh and freeze-dried PRGF eye drops were analyzed to evaluate the differential composition between both types of formulations. Furthermore, the proteomic expression of human keratocytes cells after treatment with PRGF or PRGF lyo were also analyzed to evaluate whether both formulations could induce the expression of different proteins and the differential activation of diverse signalling pathways in cells from ocular surface. Results show that, excepting a small number of proteins, the most proteins detected in PRGF and PRGF lyo are shared by both preparations (253 proteins out of 280). Furthermore, although statistical differences were observed in some deregulated proteins between PRGF and PRGF lyo (8 proteins), IPA analysis showed that these differences were not associated with the deregulation of any biological pathways. Hence, these results suggest that there are no differences between both blood-derivative products (PRGF and PRGF lyo). However, although the depletion of the most abundant proteins was carried out, the wide dynamic range of protein concentrations in blood-derived products could mask the proteins with lower concentration with respect to the most abundant proteins [37,38]. Therefore, it would be necessary to carry out a deeper analysis of scarce proteins using proteomic techniques with higher definition to unravel the possible differences in the protein composition of both products (PRGF and PRGF lyo). In fact, other authors have also revealed that metabolites within these types of formulations may present variations depending on the preparation method and the donor [39]. Therefore, the metabolome of eye drops to be used in the ocular surface should also be taken into account for future studies as these organic compounds play a mayor role in human tears. 

In the same vein, when proteomic analysis was carried out in HK cells treated with PRGF or PRGF lyo, 3213 deregulated proteins were found, of which 101 showed statistically significant differences. These significantly deregulated proteins were used to analyze the different functional processes which these proteins are involved in, identifying 41 significantly enriched GO terms. However, when IPA analysis was performed, no activation or inactivation of any cell signaling pathway was detected in relation to the significantly deregulated proteins identified among PRGF or PRGF lyo-treated HK cells. These results are in line with a previous study published by our group, where no differences in the biological activity of HK cells were observed after treatment with PRGF and PRGF lyo [26]. 

In recent years, several studies have suggested that some lyoprotectants, such as trehalose, should be added to different blood-derived products before undergoing a freeze-drying process in order to maintain their biological properties [40,41]. However, the results obtained in the present study have shown that there were no changes either in the proteomic profile of PRGF eye drops after lyophilization without the use of lyoprotectants or in the activation of different signaling pathways in HK cells compared with PRGF eye drops. These results are in accordance with those obtained in recent studies in which PRGF lyo without the use of any lyoprotectant showed no significant differences in the concentration of several growth factors and proteins and in their biological activity regarding fresh PRGF eye drops [26,27].

## 5. Conclusions

In summary, although further studies could be necessary to establish the possible proteomic differences between PRGF and PRGF lyo due to the high dynamic range of protein concentrations in these types of formulations, the present study shows that no significant differences were found in the proteomic profile between PRGF and PRGF lyo. Furthermore, these results suggest that PRGF and PRGF lyo induce similar signaling pathways activation in HK cells. All in all, the results observed in the present study suggest that no modifications are suffered by PRGF eye drops after undergoing a freeze-drying process.

## Figures and Tables

**Table 1:** Statistically significant de-regulated proteins between PRGF vs PRGF lyo.

Protein Accession Number	Gene Name	Protein Description	Fold Change	*p*-Value
P22352	GPX3	Glutathione peroxidase 3	0.7	0.0077
P08603	CFAH	Complement factor H	0.9	0.0142
P04196	HRG	Histidine-rich glycoprotein	1.1	0.0245
Q9BYE9	CDHR2	Cadherin-related family member 2	0.6	0.0252
P02749	APOH	Beta-2-glycoprotein 1	0.8	0.0336
P37802	TAGL2	Transgelin-2	0.5	0.0436
P02649	APOE	Apolipoprotein E	0.6	0.0464
Q9UHG3	PCYOX	Prenylcysteine oxidase 1	0.8	0.0488

**Table 2:** Gene Ontology analysis of de-regulated proteins with statistically significant differences between PRGF vs PRGF lyo.

GO Term	GO Definition	Genes	%	*p*-Value
0051918	Negative regulation of fibrinolysis	P02749, P04196	25	0.0042
0030195	Negative regulation of blood coagulation	P02749, P02649	25	0.0050
0043537	Negative regulation of blood vessel endothelial cell migration	P02649, P04196	25	0.0058
0001937	Negative regulation of endothelial cell proliferation	P02749, P02649	25	0.0120
0006641	Triglyceride metabolic process	P02749, P02649	25	0.0145
0000302	Response to reactive oxygen species	P02649, P22352	25	0.0161
0016525	Negative regulation of angiogenesis	P02749, P04196	25	0.0256
0098869	Cellular oxidant detoxification	P02649, P22352	25	0.0288
0030855	Epithelial cell differentiation	Q9BYE9, P37802	25	0.0288
0010468	Regulation of gene expression	P02649, P04196	25	0.0409
0002576	Platelet degranulation	P02749, P04196	25	0.0421
0006979	Response to oxidative stress	P02649, P22352	25	0.0449

## Data Availability

All the obtained data used to support the findings of this study are available from the corresponding author upon reasonable request.

## Supplementary Materials

The following supporting information can be downloaded at: https://www.mdpi.com/article/10.3390/biom12091215/s1, Supplementary Files S1–S5.
